# A note on investigating co‐occurrence patterns and dynamics for many species, with imperfect detection and a log‐linear modeling parameterization

**DOI:** 10.1002/ece3.7604

**Published:** 2021-06-06

**Authors:** Darryl I. MacKenzie, Jason V. Lombardi, Michael E. Tewes

**Affiliations:** ^1^ Proteus Outram New Zealand; ^2^ Department of Mathematics and Statistics University of Otago Dunedin New Zealand; ^3^ Caesar Kleberg Wildlife Research Institute Texas A&M University‐Kingsville Kingsville TX USA

**Keywords:** bobcat (*Lynx rufus*), coyote (*Canis latrans*), imperfect detection, log‐linear model, multiple season, ocelot (*Leopardus pardalis*), single season, species co‐occurrence

## Abstract

1. Patterns in, and the underlying dynamics of, species co‐occurrence is of interest in many ecological applications. Unaccounted for, imperfect detection of the species can lead to misleading inferences about the nature and magnitude of any interaction. A range of different parameterizations have been published that could be used with the same fundamental modeling framework that accounts for imperfect detection, although each parameterization has different advantages and disadvantages.
2. We propose a parameterization based on log‐linear modeling that does not require a species hierarchy to be defined (in terms of dominance) and enables a numerically robust approach for estimating covariate effects.
3. Conceptually, the parameterization is equivalent to using the presence of species in the current, or a previous, time period as predictor variables for the current occurrence of other species. This leads to natural, “symmetric,” interpretations of parameter estimates.
4. The parameterization can be applied to many species, in either a maximum likelihood or Bayesian estimation framework. We illustrate the method using camera‐trapping data collected on three mesocarnivore species in South Texas.

## 1 INTRODUCTION

Examining patterns of species co‐occurrence has a long history in ecology. One of the earliest examples of statistical analysis in modern‐day ecology was examining the independence of fish species in Illinois streams using a simple two‐way contingency table (Forbes, 1907). Since then, there have been a large number of publications devoted to the development, and application, of statistical methods to evaluate the level of independence of species occurrence in an area of interest (e.g., Connor & Simberloff, 1979; Diamond & Gilpin, 1982; Dice, 1945; Manly, 1995; Pielou, 1977), and investigating possible covariate relationships (e.g., Kelt et al., 1995; Peres‐Neto et al., 2001). Prior to the mid‐2000s, little attention had been devoted to the practical sampling issue of imperfect detection with species co‐occurrence assessments, that is, species may occur at a surveyed location, yet be undetected by the field methods employed (but see Cam et al., 2000). This will lead to “false absences” that may result in misleading inferences about species co‐occurrence patterns. MacKenzie et al. (2006) demonstrated that when the probability of species detection is unaffected by the presence of other species, the direction of any association between the two species (i.e., positive or negative effect on co‐occurrence) may be correctly estimated using methods that do not account for imperfect detection, but the magnitude of the dependence will be underestimated. Whereas, when detection probability of one species is different depending on the presence of the second species (e.g., due to behavioral changes in the presence of a competing species), using methods that ignore imperfect detection may not even estimate the direction of any association correctly.

MacKenzie et al. (2004) developed a modeling approach to investigate co‐occurrence patterns between two species, while accounting for imperfect detection. An important basis of their method is recognizing that with two species of interest, a surveyed location may be in one of four possible states defined by the presence or absence of each species (i.e., species *A* and *B* present, only species *A* present, only species *B* present, or neither species present). MacKenzie et al. (2004) parameterized the co‐occurrence component of their model in terms of the joint probability of both species occurring at a unit ($\psi^{\mathrm{AB}}$) and the marginal, or overall, probabilities of each species occupying a unit (i.e., $\psi^{A}$ and $\psi^{B}$). They suggested the level of co‐occurrence could be quantified in terms of:

$$\varphi =\frac{\psi^{\mathrm{AB}}}{\psi^{A}\psi^{B}},$$

where a value of 1 would imply independence. They used a similar parameterization for the detection component, noting that which species could be detected in a survey of a unit would depend on the “true” state of the location. Potential covariate relationships with any of the parameters could be explored; however, it was found to be numerically unstable because of the constraints imposed upon possible parameter values (MacKenzie et al., 2006). Richmond et al. (2010) and Waddle et al. (2010) independently implemented an alternative parameterization (hereafter referred to as the RW parameterization) of the MacKenzie et al. (2004) model that was more numerically robust, particularly with covariates. The RW parameterization requires identifying a hierarchy between species where species *A* is defined as the “dominant” species and species *B* is the “subordinate” species, where the “subordinate” species is the focal species in an analysis (i.e., how is the occurrence of species *B* affected by the presence/absence of species *A*). The model is parameterized in terms of the marginal occurrence probability of species *A* and the occurrence probability for species *B* conditional on species *A* being either present or absent from the unit (denoted here as $\psi^{B|A}$ and $\psi^{B|a}$, respectively; with the lowercase “*a”* indicating absence of species *A*). A similar conditional parameterization was also implemented for the detection component of the model. The RW parameterization could be regarded as “asymmetric” as a direction to the interaction between species is assumed, while the MacKenzie et al. (2004) parameterization is “symmetric” as no direction is assumed. While both the MacKenzie et al. (2004) and RW models were initially presented in the context of co‐occurrence between two species, they generalize to situations with a greater number of species, with the number of possible parameters to estimate increasing exponentially with the number of species (although constraints could be applied to reduce the number of parameters in the model).

Rota et al. (2016) developed a species co‐occurrence model using a “multivariate Bernoulli distribution,” which has one Bernoulli random variable per species. However, this is essentially the same general approach used by earlier authors, where possible states are defined in terms of the combinations of which species are present or absent. Therefore, the Rota et al. (2016) model can be considered as another parameterization, which, for the two‐species situation, is in terms of the conditional probabilities $\psi^{A|b}$ and $\psi^{B|a}$, and the odds ratio of co‐occurrence *v* (MacKenzie et al., 2018, p. 530). The odds ratio *v* indicates how the odds of occurrence for one species is different given the presence or absence of the other species and is the same for either species. The Rota et al. (2016) parameterization is therefore symmetric (as with the MacKenzie et al. (2004) model), with the numerical robustness of the RW parameterization.

The underlying dynamic processes of species co‐occurrence are also of interest to many ecologists, although methods to quantify them have received much less attention than those examining co‐occurrence patterns, particularly while also accounting for the imperfect detection of the target species (although see Fidino et al., 2019; Haynes et al., 2014; MacKenzie et al., 2006; Miller et al., 2012; Yackulic et al., 2014). As in the static co‐occurrence situation, there are numerous ways in which such a model could be parameterized to quantify the level of interaction between species in terms of co‐occurrence dynamics (e.g., Fidino et al., 2019; MacKenzie et al., 2006, 2018).

In this paper, we first note the link between the “multivariate Bernoulli distribution” used by Rota et al. (2016) and the well‐known statistical method of log‐linear modeling used for analyzing contingency table or count data (e.g., Poisson regression). Understanding this connection improves our ability to formulate, and interpret, models for more than two species. We also detail how a dynamic multispecies model could be defined using the log‐linear framework, with a simple example application. In the following, we focus on how the models can be parameterized in terms of log‐linear models and do not supply the details of the underlying modeling procedure, as that has been suitably described elsewhere (e.g., Fidino et al., 2019; MacKenzie et al., 2004, 2009, 2018; Richmond et al., 2010; Rota et al., 2016; Waddle et al., 2010).

## 2 MATERIALS AND METHODS

### 2.1 General sampling situation

Throughout this paper, we assume a situation where *s* sampling units (e.g., grid cells, ponds, habitat patches) have been selected from the wider population of units of interest for surveying, ideally using a probabilistic sampling scheme (to extrapolate to unsurveyed units). Units are surveyed for the presence of each species of interest, possibly at systematic points in time when co‐occurrence dynamics are of interest. At each of the *T* survey periods (that shall be referred to as *seasons* henceforth), it is assumed that the species’ distributions are static or stable; therefore, the pattern of co‐occurrence is assumed to be stable in each season. Changes in the distributions, and co‐occurrence, are allowed between seasons. Due to imperfect detection, multiple surveys of each unit are conducted each season. The number of surveys may vary spatially and temporally (MacKenzie et al., 2004, 2018).

### 2.2 Log‐linear models

Log‐linear models are used to analyze count data, particularly to assess the independence of factors used to construct contingency tables, and possibly other predictor variables. Analyses can be conducted on the counts in each cell of the table, or on the underlying cell probability structure (as done here; i.e., the probability an observation has a particular combination of factor values). It is not possible to separately estimate parameter values for all combinations of factor levels, and constraints must be applied. One option is the “corner‐point constraint” where the values for parameters associated with one row and one column are set equal to 0, with either the first, or last, row and column typically being used. For example, consider a 2 × 2 contingency table for factors *U* and *V*, and let *i* index the row and column of the table (i.e., i={u,v}, where u=1,2 and v=1,2). The log‐linear model for the cell probability $\pi_{i}$ could be defined as:

$$\log\left(\pi_{i}\right)=\alpha^{U}+\alpha^{V}+\alpha^{\mathrm{UV}}‐\log\left(K\right),$$

where *K* is a normalizing constant such that the $\pi_{i}$'s sum to 1.0. The $\alpha^{U}$parameter defines the effect of level 2 of factor *U* on the probability when v=1, the $\alpha^{V}$parameter defines the effect of level 2 of factor *V* on the probability when u=1. The $\alpha^{\mathrm{UV}}$parameter defines the level of interaction, or dependence, between factors *U* and *V* on the probability structure. The two factors are independent when $\alpha^{\mathrm{UV}}=0$, and in many applications, it is the nature of the interaction between the factors on the cell probabilities (or counts) that is of interest. The cell probabilities for a 2 × 2 table are given in more detail in Table 1, where $K=1+\exp\left(\alpha^{U}\right)+\exp\left(\alpha^{V}\right)+\exp\left(\alpha^{U}+\alpha^{V}+\alpha^{\mathrm{UV}}\right)$.

**TABLE 1:** Example of cell probability ($\pi_{i}$) structure for 2 × 2 contingency table, using the corner‐point constraint. *U* and *V* are the factors of interest, each with 2 levels. The binary indicator variables ($z_{i}^{U}$ and $z_{i}^{V}$) for the second level of each factor are also presented

*U*	*V*	$z_{i}^{U}$	$z_{i}^{V}$	$\log\left(\pi_{i}\right)$	$\pi_{i}$
1	1	0	0	$0‐\log\left(K\right)$	1/*K*
2	1	1	0	$\alpha^{U}‐\log\left(K\right)$	$\exp(\alpha^{U})/K$
1	2	0	1	$\alpha^{V}‐\log\left(K\right)$	$\exp(\alpha^{V})/K$
2	2	1	1	$\alpha^{U}+\alpha^{V}+\alpha^{\mathrm{UV}}‐\log\left(K\right)$	$\exp(\alpha^{U}+\alpha^{V}+\alpha^{\mathrm{UV}})/K$

An equivalent approach to using the corner‐point constraint is to define the log‐linear model in terms of binary indicator variables representing the levels of each factor of interest. For example, if a factor contains *M* levels, select one level to use as a reference category, then define *M*−1 binary indicator variables for observations from the other levels for that factor. In the 2 × 2 contingency table case, using the first level of factors *U* and *V* as the “reference” levels, then the indicator variables $z_{i}^{U}$ and $z_{i}^{V}$ can be defined, which equal 1 if the observed factor level was 2, and equal 0 otherwise (Table 1). The log‐linear model can then be expressed as:

$$\log\left(\pi_{i}\right)=\alpha^{U}z_{i}^{U}+\alpha^{V}z_{i}^{V}+\alpha^{\mathrm{UV}}z_{i}^{U}z_{i}^{V}‐\log\left(K\right).$$

Hence, in a regression context, the indicator variables are predictor variables representing the combination of factor levels for an observation, and the *α* terms are regression coefficients quantifying the magnitude of the effect for each factor level. Coefficients associated with an interaction between two (or more) factors, for example, the parameter $\alpha^{\mathrm{UV}}$ for the $z_{i}^{U}z_{i}^{V}$ interaction, quantify how the effect of one factor is different depending on the value of the other factor(s).

When there are more than 2 levels for a factor, then the log‐linear model generalizes in the obvious manner. For example, if factor *U* had 2 levels and factor *V* contained 3, the indicator variables $z_{i}^{V2}$ and $z_{i}^{V3}$ could be defined to equal 1 if the observed factor level was 2 or 3, respectively. The log‐linear model would then be:

$$\log\left(\pi_{i}\right)=\alpha^{U}z_{i}^{U}+\alpha^{V2}z_{i}^{V2}+\alpha^{V3}z_{i}^{V3}+\alpha^{UV2}z_{i}^{U}z_{i}^{V2}+\alpha^{UV3}z_{i}^{U}z_{i}^{V3}‐\log\left(K\right).$$

Similarly, the approach easily generalizes to a greater number of factors. For example, with three factors (*U*, *V,* and *W*) with two levels each, then:

$$\log\left(\pi_{i}\right)=\alpha^{U}z_{i}^{U}+\alpha^{V}z_{i}^{V}+\alpha^{W}z_{i}^{W}+\alpha^{\mathrm{UV}}z_{i}^{U}z_{i}^{V}+\alpha^{\mathrm{UW}}z_{i}^{U}z_{i}^{W}+\alpha^{\mathrm{VW}}z_{i}^{V}z_{i}^{W}+\alpha^{\mathrm{UVW}}z_{i}^{U}z_{i}^{V}z_{i}^{W}‐\log\left(K\right).$$

In all cases, *K* would be defined differently to ensure that the cell probabilities sum to one.

### 2.3 Species co‐occurrence data—single season

Species co‐occurrence data, assuming perfect detection, can be represented as a contingency table. Each factor is a species, and in the absence/presence case, there are two levels for each species (henceforth denoted with lowercase and uppercase characters, respectively). The structure of the possible observations for two species (species *A* and *B*), indicator variables, and associated cell probability structure is given in Table 2. The log‐linear model, expressed in terms of the indicator variables, would therefore be:

$$\log\left(\pi_{i}\right)=\alpha^{A}z_{i}^{A}+\alpha^{B}z_{i}^{B}+\alpha^{\mathrm{AB}}z_{i}^{A}z_{i}^{B}‐\log\left(K\right),$$

where $z^{A}$ and $z^{B}$ are the binary‐valued variables indicating the presence of each species. While covariates have not been considered here, the general cell probability structure is the same as that used Rota et al. (2016) where the set of indicator variables represent their “multivariate Bernoulli distribution,” with $\alpha^{A}$, $\alpha^{B},$ and $\alpha^{\mathrm{AB}}$ being equivalent to the $f_{1}$, $f_{2}$, and $f_{12}$ parameters defined by Rota et al. (2016).

**TABLE 2:** Example of cell probability ($\pi_{i}$) structure for a 2‐species (*A* and *B*) co‐occurrence application

Sp. A	Sp. B	State (*i*)	$z_{i}^{A}$	$z_{i}^{B}$	$\pi_{u,v}$
Absent	Absent	ab	0	0	1/*K*
Present	Absent	Ab	1	0	$\exp(\alpha^{A})/K$
Absent	Present	aB	0	1	$\exp(\alpha^{B})/K$
Present	Present	AB	1	1	$\exp(\alpha^{A}+\alpha^{B}+\alpha^{\mathrm{AB}})/K$

As shown by Rota et al. (2016), the model parameters are directly interpretable in terms of the probability of each species being present, conditional upon the presence or absence of the other species. That is:

$$\text{logit}\left(\psi^{A|b}\right)=\alpha^{A},$$

$$\text{logit}\left(\psi^{A|B}\right)=\alpha^{A}+\alpha^{\mathrm{AB}},$$

$$\text{logit}\left(\psi^{B|a}\right)=\alpha^{B},$$

$$\text{logit}\left(\psi^{B|A}\right)=\alpha^{B}+\alpha^{\mathrm{AB}}.$$

Therefore, $\alpha^{A}$ and $\alpha^{B}$ determine the probability of occupancy (on the logit‐scale) for each species given the absence of the other species, and $\alpha^{\mathrm{AB}}$ is the effect that the presence of one species has on the other. Hence, $\alpha^{\mathrm{AB}}$ parameter is a symmetric measure of co‐occurrence between the two species, where $\alpha^{\mathrm{AB}}=0$ indicates the species co‐occur independently, while a negative value indicate some form of exclusion or avoidance and a positive value indicate the species tend to occur together. Inferences about the level of co‐occurrence between species could be based on estimates of $\alpha^{\mathrm{AB}}$ (e.g., by considering confidence intervals), or one could “test” for independence of the species by comparing the fit of a model where $\alpha^{\mathrm{AB}}$ is estimated, to the fit of a model with the constraint $\alpha^{\mathrm{AB}}=0$. Note that the level of association can also be expressed as an odds ratio:

$$\begin{array}{cc} \nu & =\exp\left(\alpha^{\mathrm{AB}}\right)\\ & =\frac{\psi^{A|B}/\left(1‐\psi^{A|B}\right)}{\psi^{A|b}/\left(1‐\psi^{A|b}\right)}\\ & =\frac{\psi^{B|A}/\left(1‐\psi^{B|A}\right)}{\psi^{B|a}/\left(1‐\psi^{B|a}\right)}. \end{array}$$

Therefore, this is similar to the RW parameterization, but the interaction between species is modeled as a symmetric relationship.

Heuristically, the presence or absence of one species is being used as a covariate on the probability of occurrence of the other species.

The extension to more than two species is therefore straightforward. For example, with three species a third indicator variable can be defined ($z^{C}$) and the model for the contingency table cell probabilities becomes:

$$\log\left(\pi_{i}\right)=\alpha^{A}z_{i}^{A}+\alpha^{B}z_{i}^{B}+\alpha^{C}z_{i}^{C}+\alpha^{\mathrm{AB}}z_{i}^{A}z_{i}^{B}+\alpha^{\mathrm{AC}}z_{i}^{A}z_{i}^{C}+\alpha^{\mathrm{BC}}z_{i}^{B}z_{i}^{C}+\alpha^{\mathrm{ABC}}z_{i}^{A}z_{i}^{B}z_{i}^{C}‐\log\left(K\right).$$

The parameters $\alpha^{\mathrm{AB}}$, $\alpha^{\mathrm{AC}}$ and $\alpha^{\mathrm{BC}}$ quantify the two‐way interactions between species and $\alpha^{\mathrm{ABC}}$ the three‐way interaction. As noted by Rota et al. (2016), and also MacKenzie et al. (2018, p. 555), it is not always necessary to estimate higher‐order interaction terms between many species, and in fact, very large sample sizes may be required to obtain reliable parameter estimates. Furthermore, complex interactions between many species will be difficult to interpret biologically. Therefore some higher‐order interaction terms may be set equal to zero. In the log‐linear modeling literature, this is known as conditional independence. For example, the occurrence of species *A* and *B* may appear to be not independent, but that is because both species have a nonindependent co‐occurrence relationship with species *C*. Given the presence or absence of species *C*, species *A* and *B* occur independently of each other (i.e., species *A* and *B* are conditionally, upon species *C*, independent). This hypothesis could be fit by constraining $\alpha^{\mathrm{ABC}}=0$ and $\alpha^{\mathrm{AB}}=0$.

#### 2.3.1 Covariates

The effect of potential covariates on the occurrence, or co‐occurrence, for each species can be easily incorporated in the log‐linear modeling framework, where the effect of such covariates may be the same, or different for each species. For example, if a covariate $x_{1}$ is thought to affect the occurrence of species *A*, the covariate $x_{2}$ affect the occurrence of species *B*, but the level of co‐occurrence interaction is unaffected by either covariate, the following model could be fit to the data:

$$\log\left(\pi_{i}\right)=\left(\alpha^{A}+\beta_{1}^{A}x_{1}\right)z_{i}^{A}+\left(\alpha^{B}+\beta_{2}^{B}x_{2}\right)z_{i}^{B}+\alpha^{\mathrm{AB}}z_{i}^{A}z_{i}^{B}‐\log\left(K\right).$$

If covariate $x_{1}$ is also thought to affect the level of interaction between species, then another model could be fit:

$$\log\left(\pi_{i}\right)=\left(\alpha^{A}+\beta_{1}^{A}x_{1}\right)z_{i}^{A}+\left(\alpha^{B}+\beta_{2}^{B}x_{2}\right)z_{i}^{B}+\left(\alpha^{\mathrm{AB}}+\beta_{1}^{\mathrm{AB}}x_{1}\right)z_{i}^{A}z_{i}^{B}‐\log\left(K\right).$$

Interpretation of the covariate effects would proceed exactly as normal.

### 2.4 Extension to multiple seasons

To examine how species co‐occurrences change over time, it is necessary to have data from multiple seasons, preferably at equally spaced intervals. A general approach to analyzing such data is to model how the combination of species present at each unit changes over time. A transition probability matrix (TPM) can be defined that provides the probability structure for which combination of species was present in season *t* + 1, given that combination of species present at a unit in season *t* (MacKenzie et al., 2018, §14.5). For example, in the two‐species case, the TPM would be of the form:

$$\phi_{t}=\left[\begin{array}{cccc} \text{ab}\to \text{ab} & \text{ab}\to \text{Ab} & \text{ab}\to \text{aB} & \text{ab}\to \text{AB}\\ \text{Ab}\to \text{ab} & \text{Ab}\to \text{Ab} & \text{Ab}\to \text{aB} & \text{Ab}\to \text{AB}\\ \text{aB}\to \text{ab} & \text{aB}\to \text{Ab} & \text{aB}\to \text{aB} & \text{aB}\to \text{AB}\\ \text{AB}\to \text{ab} & \text{AB}\to \text{Ab} & \text{AB}\to \text{aB} & \text{AB}\to \text{AB} \end{array}\right]$$

where X→Y denotes the probability of transitioning from occupancy state X in season *t* to state Y in season *t* + 1 (where the states are denoted as above). Importantly, the elements of each row must sum to 1, as a unit must be in one of the four states by the next season. When there are *l* species of interest, then the dimension of the TPM will be $2^{l}\times 2^{l}$.

As noted by MacKenzie et al. (2018, §14.5), there are a range of possible parameterizations that could be used to estimate the parameters associated with the transition probabilities. Building on the log‐linear parameterization outlined above for the single‐season situation, the expected cell probabilities could be defined in terms of the binary indicator variables for the presence/absence of each species at both times *t* and *t* + 1 (Table 3).

**TABLE 3:** Binary variable coding for 2‐species multiseason co‐occurrence model

Row	Column	State *t*(*i*)	State *t* + 1 (*j*)	$z_{i}^{A}$	$z_{i}^{B}$	$z_{j}^{A}$	$z_{j}^{B}$
1	1	ab	ab	0	0	0	0
1	2	ab	Ab	0	0	1	0
1	3	ab	aB	0	0	0	1
1	4	ab	AB	0	0	1	1
2	1	Ab	ab	1	0	0	0
2	2	Ab	Ab	1	0	1	0
2	3	Ab	aB	1	0	0	1
2	4	Ab	AB	1	0	1	1
3	1	aB	ab	0	1	0	0
3	2	aB	Ab	0	1	1	0
3	3	aB	aB	0	1	0	1
3	4	aB	AB	0	1	1	1
4	1	AB	ab	1	1	0	0
4	2	AB	Ab	1	1	1	0
4	3	AB	aB	1	1	0	1
4	4	AB	AB	1	1	1	1

Let $z_{i}^{X}$ denotes the presence of species *X* in given state in season *t*, $z_{j}^{X}$ denotes the presence of the species in season *t* + 1. The general structure for the cell probability in row *i* and column *j* could be defined as:

$$\begin{array}{cc} \log\left(\pi_{i,j}\right) & =\beta^{A}z_{j}^{A}+\beta^{B}z_{j}^{B}+\beta^{\mathrm{AB}}z_{j}^{A}z_{j}^{B}\\ & \;+\left(\gamma^{A}z_{j}^{A}+\gamma^{B}z_{j}^{B}+\gamma^{\mathrm{AB}}z_{j}^{A}z_{j}^{B}\right)z_{i}^{A}\\ & \;+\left(\delta^{A}z_{j}^{A}+\delta^{B}z_{j}^{B}+\delta^{\mathrm{AB}}z_{j}^{A}z_{j}^{B}\right)z_{i}^{B}\\ & \;+\left(\xi^{A}z_{j}^{A}+\xi^{B}z_{j}^{B}+\xi^{\mathrm{AB}}z_{j}^{A}z_{j}^{B}\right)z_{i}^{A}z_{i}^{B}\\ & \;‐\log\left(K\right) \end{array}$$

where *K* is a normalizing constant defined to ensure the probabilities for each row of the TPM sum to 1.

This is a very general formulation, allowing complex relationships about the dynamic co‐occurrence processes to be evaluated, providing sufficient data. However, the model can be simplified by applying constraints to some parameters. For example, the γ, δ, and ξ parameters are all associated with the effects of the presence of each species in the previous season (season *t*), on which combination of species are present in the current season (season *t* + 1). This represents a situation where changes in occurrence (and co‐occurrence) can be represented as a Markov process. Constraining all these parameters to equal 0 represents a model where the probability of which species are present in season *t* + 1 is independent of the combination of species that were present in season *t* (i.e., non‐Markovian, or a random process). Alternatively, one may set only the ξ parameters to 0, representing a situation where the presence of each species in season *t* has an effect on the co‐occurrence structure in season *t* + 1, but only as additive effects. If the constraints $\beta^{\mathrm{AB}}=\gamma^{B}=\gamma^{\mathrm{AB}}=\delta^{A}=\delta^{\mathrm{AB}}=0$ are also enforced, that represents a model where the occurrence of each species changes as a Markov process, but changes are independent for each species. Finally, in the model where δ=ξ=0, the γ parameters indicate how the presence of species *A* in season *t* affects the co‐occurrence between the species in the next season. Specifically, the parameters $\gamma^{B}$ and $\gamma^{\mathrm{AB}}$ quantify what effect the presence of species *A* in season *t* has on the probability of species *B* being present in season *t* + 1. One could make a‐priori predictions about the expected direction of such effects based on whether the species are considered to exclude one another, or not.

Generalizing to a greater number of species is achieved by defining the respective set of binary indicator variables for the presence of each species in seasons *t* and *t* + 1, with potentially a large number of parameters associated with the full model (including all interaction terms among species). Regardless of whether it is possible to estimate many of those parameters for a given dataset, interpretation of the effects may be challenging. Hence, it is recommended that practitioners limit the number of interaction terms they include in a model when analyzing data and carefully consider the biological interpretation of the estimates.

### 2.5 Modeling the detection component

An important consideration for modeling the detection component is that the possible number of categories, or types of detection, will vary depending on which combination of species are present at a unit. For example, if only one species of interest is present at a unit, then there are two types of detections (nondetection/detection of that species), while if two of the target species are present there are four possible detection outcomes from a survey. This is demonstrated in Table 4 for the two‐species case. The number of possible observations can be accounted for by defining the detection component to be both a function of the true (but unknown) presence/absence of the species ($z_{i}^{X}$ indicator variables) and binary indicator variables based on the observed outcomes of each survey, which will be defined as $h_{k}^{X}$.

**TABLE 4:** Possible observations admitting imperfect detection. Lowercase characters for the true state or survey observation (Obs) indicate the absence or nondetection of that species, respectively, while uppercase characters indicate the presence or detection of that species. $z_{i}^{X}$ is the binary indicator variable for the presence or absence of species *X* and $h_{k}^{X}$ is the binary indicator variable for the detection or nondetection of species *X* in a survey

True State (*i*)	$z_{i}^{A}$	$z_{i}^{B}$	Obs (*k*)	$h_{k}^{A}$	$h_{k}^{B}$
Ab	0	0	ab	0	0
Ab	1	0	ab	0	0
Ab	1	0	Ab	1	0
aB	0	1	ab	0	0
aB	0	1	aB	0	1
AB	1	1	ab	0	0
AB	1	1	Ab	1	0
AB	1	1	aB	0	1
AB	1	1	AB	1	1

Detection probability can therefore be defined using a log‐linear modeling framework as:

$$\begin{array}{cc} \log\left(p_{i,k}\right) & =\eta^{A}h_{k}^{A}z_{i}^{A}\\ & \;+\eta^{B}h_{k}^{B}z_{i}^{B}\\ & \;+\left(\zeta^{A}h_{k}^{A}+\zeta^{B}h_{k}^{B}+\zeta^{\mathrm{AB}}h_{k}^{A}h_{k}^{B}\right)z_{i}^{A}z_{i}^{B}\\ & \;‐\log\left(K\right) \end{array}$$

where,

$$\begin{array}{cc} K & =1+\left(\exp\left(\eta_{1}\right)\right)z_{i}^{A}\left(1‐z_{i}^{B}\right)\\ & \;+\left(\exp\left(\eta_{2}\right)\right)\left(1‐z_{i}^{A}\right)z_{i}^{B}\\ & \;+\left(\exp\left(\eta_{1}+\eta_{3}\right)+\exp\left(\eta_{2}+\eta_{4}\right)+\exp\left(\eta_{1}+\eta_{2}+\eta_{3}+\eta_{4}+\eta_{5}\right)\right)z_{i}^{A}z_{i}^{B}. \end{array}$$

### 2.6 Example—mesocarnivores in Texas

The motivation for developing this parameterization of the multiseason co‐occurrence model was a 7‐year camera trap dataset of bobcats (*Lynx rufus*), ocelot (*Leopardus pardalis*), and coyote (*Canis latrans*) collected in South Texas (Lombardi et al., 2020). This dataset is part of a long‐term ocelot monitoring study on the East Foundation's El Sauz Ranch in Willacy and Kenedy counties, Texas. Although ocelot share a geographic overlap with bobcats and coyotes from South Texas to Central Mexico (Hody & Kays, 2018; Horne et al., 2009; Sánchez‐Cordero et al., 2008), interactions among this community are poorly understood in this region. Coyote interactions with bobcats are well studied across their shared geographic range and often do not exhibit spatial or temporal segregation (Lesmeister et al., 2015; Thornton et al., 2004). Studies on bobcat–ocelot interactions have indicated the two species share a dietary overlap (Booth‐Binczik et al., 2013), temporally segregate movement rates (Leonard et al., 2020), and may exhibit resource partitioning at the shrub level (Horne et al., 2009). Ocelots and coyote interactions are poorly known, with co‐occurrence likely facilitated by high availability of food resources and abundant cover (Lombardi et al., 2020).

From 8 May 2011 to 24 March 2018, 56 camera traps (Cuddeback^®^ white‐flash Expert Scouting Cameras and Cuddeback^®^ X‐Change Color cameras (NonTypical, Isanti, WI, USA)) were deployed at 28 paired camera stations in the northwestern and southwestern regions of the El Sauz Ranch. Camera traps were set in forests containing live oak (*Quercus virginiana*), honey mesquite (*Prosopis glandulosa*), and thornshrub (lime prickly ash [*Zanthoxylum fagara*], huisache [*Acacia farnesiana*], and spiny hackberry [*Celtis pallida*]). Camera stations were spaced 1 km apart, which was based on the mean minimum distance moved for ocelots in the region (Lombardi et al., 2020). At a station, cameras were placed facing each other and offset 1–2 m, with each camera attached to a tree or wooden stake about 30 cm above the ground. Camera stations were maintained all year, and cameras were replaced if they malfunctioned (Lombardi et al., 2020).

A sampling season was defined to be a 20‐week period, either 8 May to 23 September (hot season) or 8 November to 24 March (cool season). A survey was defined to be a 4‐week period, that is, a species was detected ($h_{k}^{X}=1$) if it was photographed at least once at a station during the 4‐week period, and undetected ($h_{k}^{X}=0$) otherwise. Hence, each season was comprised of 5 surveys. Surveys were defined to be a 4‐week period such that detections of bobcats and coyotes within a survey period could be assumed independent (Lombardi et al., 2020, i.e., aggregating the camera data at a temporal scale of 4 weeks effectively removes the effect of any short‐term behavioral interactions between species).

The log‐linear parameterization discussed above provides a great deal of flexibility for examining the patterns and dynamics of co‐occurrence between multiple species, especially given the ability to incorporate spatial and temporal covariates. However, given the number of camera stations deployed (i.e., 28 surveyed units), only relatively simple models are fit to the data here to illustrate some key concepts. Lombardi et al. (2020) conduct a fuller analysis of the dataset examining the effect of covariates.

Due to the lack of hunting pressure (for coyotes and bobcats) in the area, we expected a natural dynamic between the three species and defined overarching hypotheses: (a) probability of ocelot and bobcat occurrence and detection will be negatively influenced by the presence/detection of coyotes, (b) ocelot and bobcat will exhibit positive co‐occurrence values, and (c) the presence of species will be influenced by the presence of another the previous season. Five models were fit to the dataset, each representing a different set of hypotheses about co‐occurrence patterns and dynamics (Table 5). While model parameters could be season‐specific, they have been assumed to be season invariant. Additional information about the exact parameterization is supplied in the Supplemental Material. The same detection component was assumed for all models, where a separate detection probability was estimated for each species, which was assumed to be independent of both the presence and detection of other species. Model 1 assumes species occur near camera trap stations independently of each other, and the probability of occurrence is the same each season and independent of the species being present near a station in the previous season. Model 3 also assumes species occur independently of each other, although the probability of occurrence after season 1 depends on the presence of the species in the previous season. This is equivalent to modeling the occurrence of each species as independent single‐species multiseason models (MacKenzie et al., 2003), where changes in occurrence are assumed to be a first‐order Markov process.

**TABLE 5:** Summary of effects included in each model fit to the Texas camera‐trapping data. “2‐way interaction” is interaction effects between pairs of species; “Depends on $z_{i}^{X}$” and “Depends on $z_{i}^{Y}"$ indicate whether occurrence in the current season depends on the presence of the focal (*X*), or other (*Y*) species in the previous season

Model	2‐way Interactions	Depends on $z_{i}^{X}$	Depends on $z_{i}^{Y}$
1	N	N	N
2	Y	N	N
3	N	Y	N
4	Y	Y	N
5	Y	Y	Y

The species co‐occurrence models were fit using maximum likelihood techniques (e.g., MacKenzie et al., 2004, 2009, 2018; Richmond et al., 2010; Waddle et al., 2010) using custom‐written R code, although Bayesian methods could also be used (e.g., Fidino et al., 2019; Rota et al., 2016). Models were compared on the basis of Akaike's information criterion (AIC).

## 3 RESULTS

### 3.1 Example—mesocarnivores in Texas

Table 6 presents a summary of the five models fit to the mesocarnivore data. On the basis of AIC, Model 4 had the majority of the support with 79% of the AIC model weight, and Model 5 also has some support with 21% AIC model weight. The results provide strong evidence that the probability of a species occurring near a station is dependent on the presence of the species near the station in the previous seasons (given ranking of Models 3–5), and affected by the presence of other species in the same season (Models 4 and 5 ranked highest). There is some indication that occurrence may also depend on the presence of other species in the previous season (Model 5 ranked second).

**TABLE 6:** Summary of the model comparison process, including the relative difference in AIC (ΔAIC), AIC model weight (*w*), number of estimated parameters (*K*), and two times the negative log‐likelihood value (*−2ll*).

Model	ΔAIC	*w*	*K*	*−2ll*
1	175.20	0.00	6	6,298.15
2	66.11	0.00	9	6,183.06
3	104.62	0.00	12	6,215.57
4	0.00	0.79	15	6,104.95
5	2.65	0.21	21	6,095.59

From Model 4, the estimated probability of detecting ocelots, bobcats, and coyotes during 4 weeks of camera trapping was estimated to be 0.43 (0.02), 0.49 (0.01), and 0.51 (0.01), respectively (standard error in parentheses). For each of the three species, the probability of occurrence in the current season is estimated to be higher if they were present in the previous season, particularly for ocelots, although the effect is small for bobcats (Table 7; parameters $\gamma^{O}$, $\delta^{B}$, and $\xi^{C}$). Note that under the parameterization used here, the *β* parameters determine the probability of occurrence given the absence of the species in the previous season, that is, the probability of colonization. Therefore, the $\gamma^{O}$, $\delta^{B}$, and $\xi^{C}$ parameters are the difference between the colonization and persistence probabilities (on the logit‐scale) for the respective species. The estimated 2‐way interaction terms (parameters $\alpha^{\mathrm{OB}}$, $\alpha^{\mathrm{OC}}$, and $\alpha^{\mathrm{BC}}$) are all positive, indicating that if one species is present, the other species are more likely to be also present. The odds ratio for the co‐occurrence of ocelots and bobcats is estimated to be 4.16, 5.31 for ocelots and coyotes, and 5.88 for bobcats and coyotes. The confidence intervals for each of the odds ratios are relatively wide, which is a reflection of the number of surveyed stations, although the intervals are all greater than 1.0 suggesting strong evidence of a positive correlation.

**TABLE 7:** Parameter estimates from Model 4 including associated standard errors, estimated odds ratio (OR) with associated lower and upper limits of 95% confidence intervals

Parameter	Est	SE	OR	Lower	Upper
$\alpha^{O}$	−2.10	0.63	0.12	0.04	0.42
$\alpha^{B}$	−1.16	0.55	0.31	0.11	0.92
$\alpha^{C}$	−1.29	0.56	0.28	0.09	0.82
$\alpha^{\mathrm{OB}}$	1.43	0.37	4.16	2.03	8.53
$\alpha^{\mathrm{OC}}$	1.67	0.48	5.31	2.07	13.60
$\alpha^{\mathrm{BC}}$	1.77	0.34	5.88	2.99	11.56
$\beta^{O}$	−3.72	0.51	0.02	0.01	0.07
$\beta^{B}$	−0.89	0.34	0.41	0.21	0.81
$\beta^{C}$	−0.56	0.34	0.57	0.30	1.11
$\gamma^{O}$	2.11	0.30	8.24	4.62	14.69
$\delta^{B}$	0.06	0.31	1.06	0.57	1.96
$\xi^{C}$	0.55	0.36	1.74	0.87	3.49

## 4 DISCUSSION

The log‐linear parameterization outlined here for the multiseason, multispecies co‐occurrence model is not unique, and other parameterizations are possible (e.g., Fidino et al., 2019; MacKenzie et al., 2006, 2018). The log‐linear parameterization provides the ability to directly estimate, and interpret, how the presence of species is affected by the presence of other species in either the current, or the previous, season. With this structure, the presence of each species is essentially being used as a predictor variable for the presence of other species, although the general framework that accounts for imperfect detection allows for the fact that the presence of any species may not be known with certainty. Furthermore, the parameterization can also be applied to the detection process, to allow for nonindependent detections of each species.

Complexity breeds complexity. As practitioners attempt to address more complex questions of ecological data, more complex methods of analysis are generally required to provide quantitative inspections of those data. Such is the case with multiseason, multispecies co‐occurrence models. Irrespective of the preferred parameterization to be used, proper analysis should involve careful consideration of hypotheses of interest, which species interactions should be included and whether such interactions change over time, effect of potential covariates for co‐occurrence‐ and detection‐related parameters. Proper analysis will require time, and some degree of skill in fitting and interpreting model results. While tools can be developed to simplify certain aspects of the process, practitioners should have a realistic expectation that such analyses require a substantial investment of time and effort.

Practitioners are strongly encouraged to gain a realistic expectation of the type, and quantity, of data required to achieve their objectives, before embarking on any data collection. Complex models, with a large number of biologically relevant parameters to estimate, will require relatively large datasets to produce accurate estimates with suitable levels of precision. Simulation studies are an incredibly useful approach to evaluating the expected quality of the results from a proposed study design. The outcome will often be enlightening, and sometimes, sobering. While the exact outcome will depend on the specifics of the situation, in general we suggest that typically the number of sampling units required to be surveyed will be in the 100's rather than the 10's of units. This is based on our experience with similar models, and on the simple premise that there is not a lot of information in binary observations, and therefore, a large number of them tend to be required to obtain adequate precision of parameter estimates.

Log‐linear modeling can be used in situations where a factor of interest has *m* levels (with m≥2), by defining m‐1 indicator variables. In this paper, we have focused on situations where m=2 (i.e., species presence or absence), although as alluded to above, this parameterization extends naturally to situations where the occurrence of species may be defined using a greater number of categories (e.g., absent, present without breeding, present with breeding). The log‐linear modeling parameterization therefore provides a framework for assessing relevant questions about co‐occurrence patterns and dynamics for these more complex situations, in combination with multistate occupancy models (e.g., MacKenzie et al., 2009; Nichols et al., 2007; Royle & Link, 2005).

This parameterization of a many‐species co‐occurrence model is currently being incorporated into Program PRESENCE and the RPresence R package. The data and R code used for the mesocarnivore example are available from the Dryad repository https://doi.org/10.5061/dryad.59zw3r26t.

## CONFLICT OF INTEREST

None declared.

## AUTHOR CONTRIBUTIONS

**Darryl I. MacKenzie:** Conceptualization (lead); Formal analysis (lead); Methodology (lead); Software (lead); Writing—original draft (lead); Writing—review and editing (lead). **Jason V. Lombardi:** Conceptualization (supporting); Data curation (lead); Writing—original draft (supporting); Writing—review and editing (supporting). **Michael E. Tewes:** Conceptualization (supporting); Supervision (supporting); Writing—review and editing (supporting).

## Data Availability

Data and associated R code used for the example in this manuscript are accessible in the repository Dryad. Please see https://doi.org/10.5061/dryad.59zw3r26t.
